# Supplementary material for: Brain structure and working memory adaptations associated with maturation and aging in mice
Source: Front Aging Neurosci. 2023 Jul 6;15:1195748. doi: 10.3389/fnagi.2023.1195748 (PMC10359104; doi:10.3389/fnagi.2023.1195748)
Supplement: Supplementary file 2 [file Table_2.DOCX]

**Supplementary Table 1. Pairwise group differences in degree in apriori regions**

|  | **Young vs. Middle-age** | | | **Middle-age vs. Old** | | |
| --- | --- | --- | --- | --- | --- | --- |
|  | **ACC** | **HPC** | **ORB** | **ACC** | **HPC** | **ORB** |
| **Threshold** | **p** | **p** | **p** | **p** | **p** | **p** |
| 0.05 | 0.245 | 1.000 | 0.210 | 0.742 | 0.785 | 0.409 |
| 0.06 | 0.257 | 1.000 | 0.282 | 0.342 | 0.433 | 0.212 |
| 0.07 | 0.106 | 0.248 | 0.328 | 0.373 | 0.474 | 0.257 |
| 0.08 | **0.042** | **0.010** | 0.607 | 0.384 | 1.000 | 0.319 |
| 0.09 | **0.013** | **0.013** | 0.633 | 0.163 | 1.000 | 0.353 |
| 0.10 | **0.018** | **0.024** | 0.640 | 0.179 | 0.838 | 0.382 |
| 0.11 | 0.068 | **0.031** | 0.661 | 0.437 | 0.551 | 0.417 |
| 0.12 | **0.078** | **0.019** | 0.675 | 0.207 | 0.562 | 0.442 |
| 0.13 | **0.039** | **0.022** | 0.494 | 0.232 | 0.574 | 0.278 |
| 0.14 | **0.044** | **0.023** | 0.357 | 0.244 | 0.372 | 0.150 |
| 0.15 | **0.049** | **0.036** | 0.709 | 0.109 | 0.398 | 0.183 |
| 0.16 | 0.052 | **0.046** | 0.708 | 0.120 | 0.408 | 0.198 |
| 0.17 | 0.123 | 0.067 | 0.723 | 0.285 | 0.138 | 0.218 |
| 0.18 | 0.062 | 0.063 | 0.732 | 0.312 | 0.142 | 0.239 |
| 0.19 | **0.023** | 0.174 | 0.720 | 0.305 | 0.144 | 0.354 |
| 0.20 | **0.031** | 0.185 | 0.912 | 0.168 | 0.156 | 0.366 |
| 0.21 | 0.069 | 0.210 | 0.579 | 0.556 | 0.152 | 0.171 |
| 0.22 | 0.065 | 0.155 | 0.574 | 0.561 | 0.271 | 0.178 |
| 0.23 | **0.028** | 0.168 | 0.573 | 0.578 | 0.273 | 0.194 |
| 0.24 | **0.021** | 0.186 | 0.589 | 0.570 | 0.436 | 0.204 |
| 0.25 | **0.002** | 0.219 | 0.909 | 0.582 | 0.641 | 0.218 |
| 0.26 | **0.002** | 0.157 | 1.000 | 0.579 | 0.871 | 0.218 |
| 0.27 | **0.001** | 0.063 | 0.910 | 0.865 | 0.874 | 0.151 |
| 0.28 | **0.000** | 0.071 | 0.917 | 1.000 | 0.865 | 0.144 |
| 0.29 | **0.000** | 0.086 | 0.902 | 1.000 | 0.868 | 0.161 |
| 0.30 | **0.001** | 0.157 | 0.908 | 0.878 | 0.874 | 0.104 |
| 0.31 | **0.004** | 0.254 | 0.897 | 0.858 | 0.867 | 0.331 |
| 0.32 | **0.004** | 0.395 | 1.000 | 0.869 | 0.870 | 0.425 |
| 0.33 | **0.001** | 0.410 | 1.000 | 0.871 | 1.000 | 0.432 |
| 0.34 | **0.003** | 0.426 | 1.000 | 0.868 | 0.861 | 0.446 |
| 0.35 | **0.003** | 0.415 | 0.897 | 1.000 | 0.861 | 0.584 |
| 0.36 | **0.003** | 0.436 | 0.885 | 1.000 | 0.862 | 0.363 |
| 0.37 | **0.003** | 0.427 | 0.834 | 1.000 | 0.366 | 0.270 |
| 0.38 | **0.012** | 0.594 | 0.650 | 0.860 | 0.369 | 0.267 |
| 0.39 | **0.010** | 0.735 | 0.651 | 1.000 | 0.373 | 0.359 |
| 0.40 | **0.010** | 0.917 | 0.881 | 0.855 | 0.198 | 0.366 |

P values corresponding to group differences in degree at each density threshold and across density thresholds. *ACC = anterior cingulate area; HPC = hippocampal formation; ORB = orbital area*
