# Supplementary material for: Brain structure and working memory adaptations associated with maturation and aging in mice
Source: Front Aging Neurosci. 2023 Jul 6;15:1195748. doi: 10.3389/fnagi.2023.1195748 (PMC10359104; doi:10.3389/fnagi.2023.1195748)
Supplement: Supplementary file 3 [file Table_3.DOCX]

**Supplementary Table 2. Apriori pairwise group differences in global SCN measures**

|  | **T1: Young vs. Middle-age** | | | **T2: Middle-age vs. Old** | | |
| --- | --- | --- | --- | --- | --- | --- |
|  | **Modularity** | **Transitivity** | **Mean Dist.** | **Modularity** | **Transitivity** | **Mean Dist.** |
| **Threshold** | **p** | **p** | **p** | **p** | **p** | **p** |
| 0.05 | 0.124 | 0.374 | 0.951 | **0.010** | 0.126 | 0.252 |
| 0.06 | 0.237 | 0.875 | **0.002** | **0.016** | 0.719 | 0.449 |
| 0.07 | 0.667 | 0.672 | **0.001** | 0.080 | 0.332 | 0.274 |
| 0.08 | 0.717 | 0.937 | 0.748 | 0.217 | 0.155 | 0.460 |
| 0.09 | 0.411 | 0.992 | 0.134 | 0.757 | 0.486 | 0.613 |
| 0.10 | 0.564 | 0.797 | 0.480 | 0.712 | 0.464 | 0.831 |
| 0.11 | 0.453 | 0.401 | 0.432 | 0.665 | 0.968 | 0.864 |
| 0.12 | 0.207 | 0.624 | 0.267 | 0.869 | 0.743 | 0.877 |
| 0.13 | 0.274 | 0.600 | 0.963 | 0.929 | 0.978 | 0.753 |
| 0.14 | 0.419 | 0.701 | 0.280 | 0.633 | 0.911 | 0.543 |
| 0.15 | 0.493 | 0.735 | 0.620 | 0.860 | 0.922 | 0.484 |
| 0.16 | **0.027** | 0.446 | 0.484 | 0.303 | 0.527 | 0.289 |
| 0.17 | **0.020** | 0.650 | 0.511 | 0.393 | 0.704 | 0.440 |
| 0.18 | **0.001** | 0.610 | 0.710 | 0.376 | 0.802 | 0.963 |
| 0.19 | 0.140 | 0.606 | 0.598 | 0.639 | 0.917 | 0.996 |
| 0.20 | 0.158 | 0.628 | 0.635 | 0.800 | 0.898 | 0.927 |
| 0.21 | 0.240 | 0.384 | 0.647 | 0.970 | 0.711 | 0.818 |
| 0.22 | 0.459 | 0.419 | 0.734 | 0.522 | 0.428 | 0.655 |
| 0.23 | 0.121 | 0.358 | 0.766 | 0.487 | 0.463 | 0.585 |
| 0.24 | **0.021** | 0.339 | 0.810 | 0.740 | 0.568 | 0.710 |
| 0.25 | **0.025** | 0.412 | 0.860 | 0.980 | 0.529 | 0.485 |
| 0.26 | **0.003** | 0.331 | 0.850 | 0.781 | 0.361 | 0.303 |
| 0.27 | **0.030** | 0.266 | 0.856 | 0.759 | 0.321 | 0.261 |
| 0.28 | **0.020** | 0.292 | 0.845 | 0.611 | 0.252 | 0.301 |
| 0.29 | **0.019** | 0.251 | 0.843 | 0.419 | 0.294 | 0.475 |
| 0.30 | 0.058 | 0.281 | 0.852 | 0.962 | 0.443 | 0.606 |
| 0.31 | **0.041** | 0.307 | 0.881 | 0.425 | 0.377 | 0.484 |
| 0.32 | 0.215 | 0.295 | 0.856 | 0.831 | 0.405 | 0.540 |
| 0.33 | 0.231 | 0.324 | 0.864 | 0.639 | 0.511 | 0.611 |
| 0.34 | 0.214 | 0.260 | 0.834 | 0.386 | 0.484 | 0.511 |
| 0.35 | 0.231 | 0.272 | 0.820 | 0.478 | 0.617 | 0.529 |
| 0.36 | 0.146 | 0.288 | 0.821 | 0.568 | 0.706 | 0.605 |
| 0.37 | 0.072 | 0.297 | 0.806 | 0.890 | 0.730 | 0.612 |
| 0.38 | 0.110 | 0.331 | 0.851 | 0.840 | 0.550 | 0.688 |
| 0.39 | 0.437 | 0.301 | 0.818 | 0.949 | 0.447 | 0.720 |
| 0.40 | 0.158 | 0.301 | 0.806 | 0.331 | 0.456 | 0.725 |

P values corresponding to group differences in global measures of structural covariance networks *(modularity, transitivity, mean distance)* at each density threshold and across density thresholds.
